# Construction of a nomogram to predict the prognosis of non-small-cell lung cancer with brain metastases

**DOI:** 10.1097/MD.0000000000021339

**Published:** 2020-07-31

**Authors:** Zhangheng Huang, Chuan Hu, Yuexin Tong, Zhiyi Fan, Chengliang Zhao

**Affiliations:** a Department of Minimally Invasive Spine Surgery, Affiliated Hospital of Chengde Medical University, Chengde, Hebei; b Department of Orthopedic, The Affiliated Hospital of Qingdao University, Qingdao, Shandong, China.

**Keywords:** brain metastases, nomogram, non-small-cell lung cancer, overall survival, SEER

## Abstract

Patients with non-small-cell lung cancer (NSCLC) often have a poor prognosis when brain metastases (BM) occur. This study aimed to evaluate the prognostic factors of BM in newly diagnosed NSCLC patients and construct a nomogram to predict the overall survival (OS).

We included NSCLC patients with BM newly diagnosed from 2010 to 2015 in Surveillance, Epidemiology, and End Results database. The independent prognostic factors for NSCLC with BM were determined by Cox proportional hazards regression analysis. We then constructed and validated a nomogram to predict the OS of NSCLC with BM.

We finally included 4129 NSCLC patients with BM for analysis. Age, race, sex, liver metastasis, primary site, histologic type, grade, bone metastasis, T stage, N stage, surgery, chemotherapy, and lung metastasis were identified as the prognostic factors for NSCLC with BM and integrated to establish the nomogram. The calibration, receiver operating characteristic curve, and decision curve analyses also showed that the clinical prediction model performed satisfactorily in predicting prognosis.

A clinical prediction model was constructed and validated to predict individual OS for NSCLC with BM. The establishment of this clinical prediction model has great significance for clinicians and individuals.

## 1 Introduction

Lung cancer (LC) is the most common cancer and the main cause of death.^[1]^ Non-small-cell lung cancer (NSCLC) is the most common in LC, accounting for about 80% to 90%.^[2,3]^ Of these, approximately 10% of NSCLC patients are diagnosed with brain metastases (BM) when they are first discovered, and 20% to 40% will develop BM at some point .^[4,5]^ Due to the limitations of treatment methods, the median survival of patients with BM is only 3 to 6 months.^[6,7]^ As such, the prevention of BM is of great significance for patients with NSCLC.

The TNM staging system is widely used clinically to predict the prognosis of LC.^[8]^ Unfortunately, the TNM staging system does not sufficiently cover cancer biology and predict the overall survival (OS) for all subtypes of lung cancer. Besides, recent research has shown that the OS of NSCLC patients in the same TNM stage are various, which indicated that other factors such as gender, race, and insurance status can also affect the prognosis of NSCLC.^[9]^ Moreover, it has been reported that the prognosis of patients varies with the organ to which the tumor has metastasized.^[10,11]^ Therefore, it is still difficult to accurately predict the OS of NSCLC with BM by using this tool.

In recent decades, nomograms have been developed to predict the prognosis of various cancers and have shown higher accuracy than the TNM staging system.^[12,13]^ Based on multifactor regression analysis, the nomogram combines multiple predictors with intuitive graphs to make the results more accessible and facilitate the evaluation of the prognosis of patients.^[11]^ Zhang et al constructed a prediction model for predicting the risk of BM in patients with NSCLC. There are also some nomograms based on big data analysis to predict the OS of NSCLC. However, until now, no studies have been conducted to establish a nomogram to predict the OS of NSCLC with BM. Thus, the purpose of this study is to construct a clinical prediction model to predict the prognosis of NSCLC with BM, hoping to provide accurate predictions.

## 2 Material and methods

### 2.1 Patients

We included NSCLC cases with BM in the Surveillance, Epidemiology and End Results (SEER) database from 2010 to 2015. Since the information of patients in the SEER database is publicly available online, this research does not require approval from our Institutional Review Board. The inclusion criteria for selecting patients were as follows: The patient was diagnosed with LC by pathological examination (primary site code: C34.1, C34.2, C34.3, C34.8); patients whose histologic type is NSCLC (histologic type code: 8140, 8141,8144, 8244, 8250–8255, 8260,8290, 8310, 8323, 8333, 8470, 8480, 8481, 8490, 8507, 8550, 8551, 8570, 8571, 8574, 8576, 8052, 8070–8075, 8083, 8084, 8123, 8004, 8012–8014, 8022, 8030–8035, 8046, 8082, 8200, 8240, 8249, 8430, 8560, 8562); patient with newly diagnosed BM; LC is the first primary malignant tumor. Patients were excluded if: patients with 2 or more primary malignancies; patients without survival date; patients missing important detailed information, including race, primary site, grade, marital status, radiotherapy, insurance status, chemotherapy, or surgery. Finally, 4129 NSCLC cases diagnosed with BM were included in this research.

### 2.2 Data elements

We extracted factors that might be associated with prognosis, including age, gender, race, primary site, histologic type, grade, laterality, T stage, N stage, surgery, radiotherapy, chemotherapy, bone metastasis, liver metastasis, lung metastasis, marital, and insurance status. The main endpoint of our study was OS, which was defined as the time interval between the day of diagnosis to death due to any cause.

### 2.3 Statistical analysis

All NSCLC patients with BM were randomly divided into the training (n = 2893) and validation (n = 1236) cohorts with a ratio of 7:3. The best cutoff value of age for OS was determined by X-tile software, and patients were segmented into 3 subgroups. In the training cohort, the variables related to prognosis were determined by the univariate Cox analysis. Then, the independent prognostic factors for NSCLC with BM were identified by multivariate Cox analysis. According to the results of the multivariate Cox analysis, the independent prognostic factors were incorporated to develop a nomogram to predict the OS for NSCLC with BM. Additionally, the curve was plotted, and the area under the time-dependent receiver operating characteristic (ROC) was used to estimate the discrimination of the clinical predictive model. Meanwhile, the calibration curves and decision curve analyses (DCA) of 1, 2, and 3 years were constructed to estimate the nomogram. All statistical analyses in this study were performed by using R software (version 3.6.1). A *P* value of less than .05 was considered as statistical significance.

## 3 Result

### 3.1 Clinicopathological characteristics of the patients

Based on our criteria, 4129 cases were enrolled from the SEER database. Then, all patients with BM were randomly classified into 2 groups. The difference between the 2 cohorts was not significantly different. In the training cohort, 50.5% of the patients were under 66 years old and 80.8% were White. In terms of tumor characteristics, BM patients often had grade III (65.1%), N2 stage (46.6%) and adenocarcinoma (70.5%), and 63.0% of the tumor occurred in the upper lobe. Regarding treatment, the vast majority of patients had not undergone surgery (91.9%), but most had undergone chemotherapy (64.4%) and radiation therapy (83.5%). Table 1  displays detailed information on demographic and clinicopathological characteristics of NSCLC with BM.

**Table 1:**
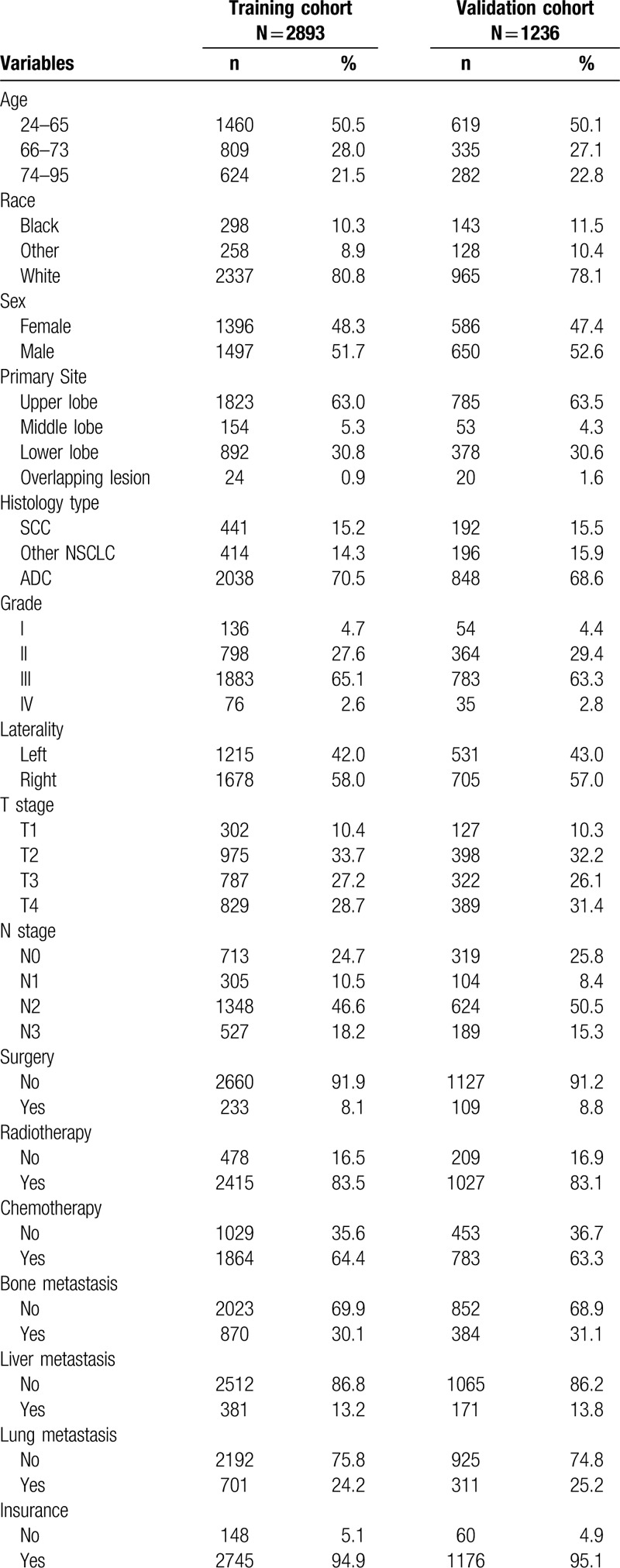
Demographic and clinicopathological characteristics of the training cohort and validation cohort.

**Table 1 (Continued):**
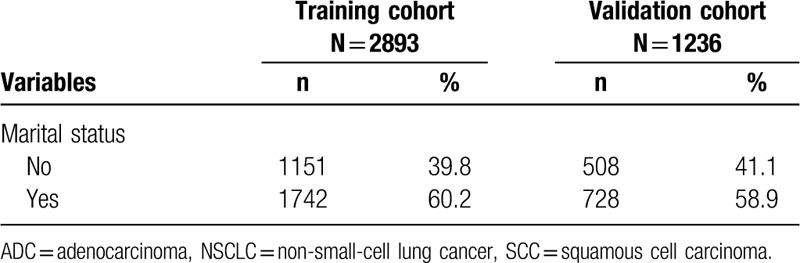
Demographic and clinicopathological characteristics of the training cohort and validation cohort.

### 3.2 Survival analysis for different numbers of metastasis sites

**Figure 1:**
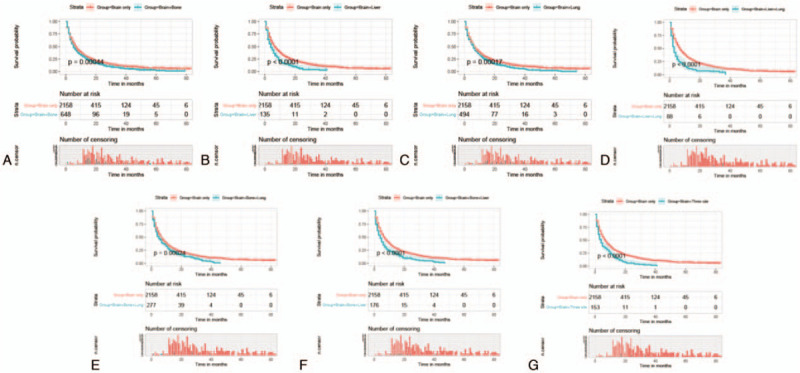
Kaplan–Meier survival analysis for different numbers of metastasis sites.

As shown in Fig. 1, there was a statistically significant difference in survival between the subgroups (*P* < .001), which implied that the numbers of metastasis sites had a significant effect on survival outcome for NSCLC with BM. In addition, patients with BM associated with multiple extracerebral metastases showed a worse prognosis than patients with single BM.

### 3.3 Prognostic factors of OS

Univariate Cox analysis was performed for the following variables: age, gender, race, primary site, histologic type, grade, laterality, T stage, N stage, surgery, radiotherapy, chemotherapy, bone metastasis, liver metastasis, lung metastasis, marital, and insurance status. The results of the univariate Cox analysis showed that age, gender, race, primary site, histologic type, grade, T stage, N stage, surgery, radiotherapy, chemotherapy, bone metastasis, liver metastasis, lung metastasis, and marital status were prognostic factors for BM in NSCLC patients (Table 2 ). These prognostic factors were subsequently included in a multivariate Cox analysis. Finally, 13 factors other than radiotherapy and marital status were identified as independent prognostic factors (Table 2 ).

**Table 2:**
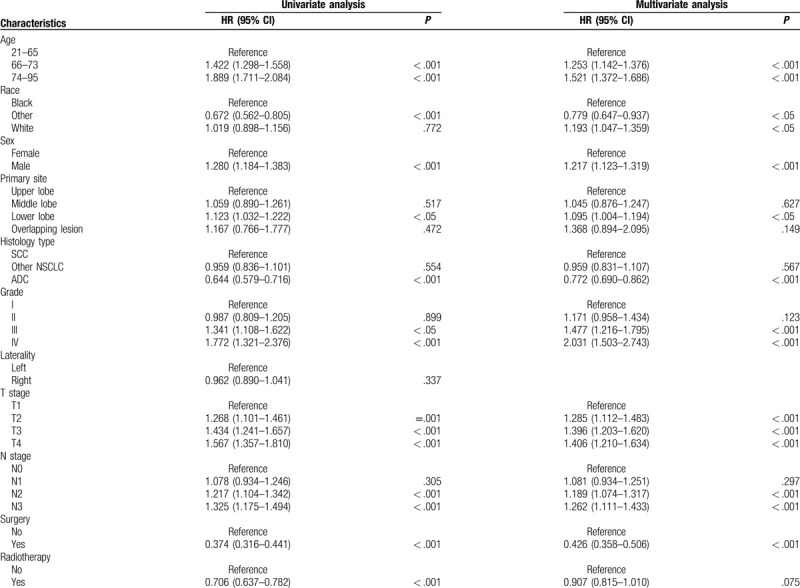
Univariate and multivariate Cox proportional hazards regression analysis of prognosis of NSCLC patients with BM.

**Table 2 (Continued):**
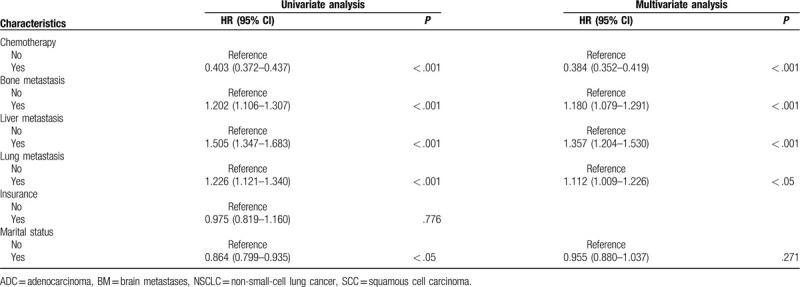
Univariate and multivariate Cox proportional hazards regression analysis of prognosis of NSCLC patients with BM.

### 3.4 Prognostic nomogram

**Figure 2:**
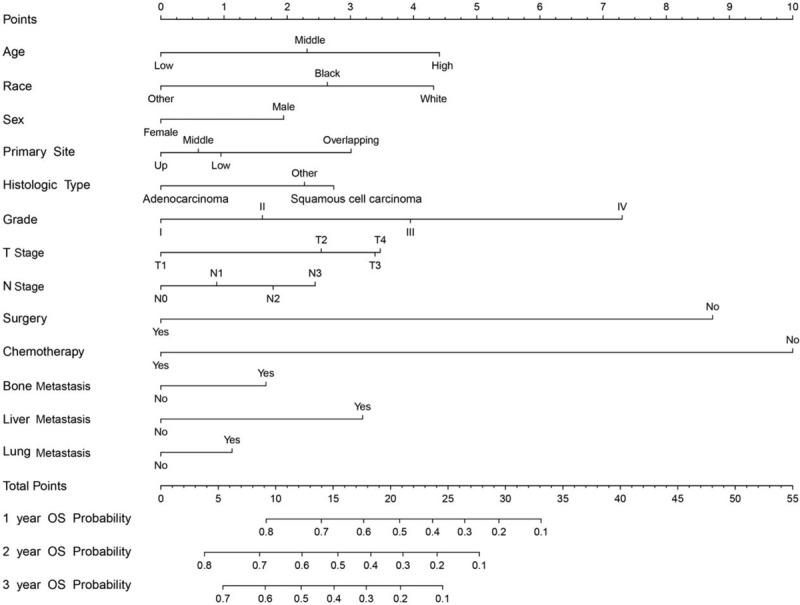
Nomogram predicting 1-year, 2-year, and 3-year OS. The total points were calculated by adding the points of each prognostic factor, and correspond to the possibilities of 1-year, 2-year, and 3-year OS of NSCLC patients with BM. BM = brain metastases, NSCLC = non-small-cell lung cancer, OS = overall survival.

According to the important prognostic factors selected in the training cohort, we developed a nomogram to predict the prognosis of BM with NSCLC (Fig. 2). Interestingly, as shown in Fig. 2, chemotherapy contributed the maximal to prognosis, followed by surgery. grade, age, race, T stage, presence or absence of liver metastasis, primary site, histologic type, and N stage, which made moderate effects on prognosis, while gender, presence or absence of bone, lung metastasis showed little effect on prognosis.

### 3.5 Comparison of nomogram and single independent prognostic factor prediction accuracy

**Figure 3:**
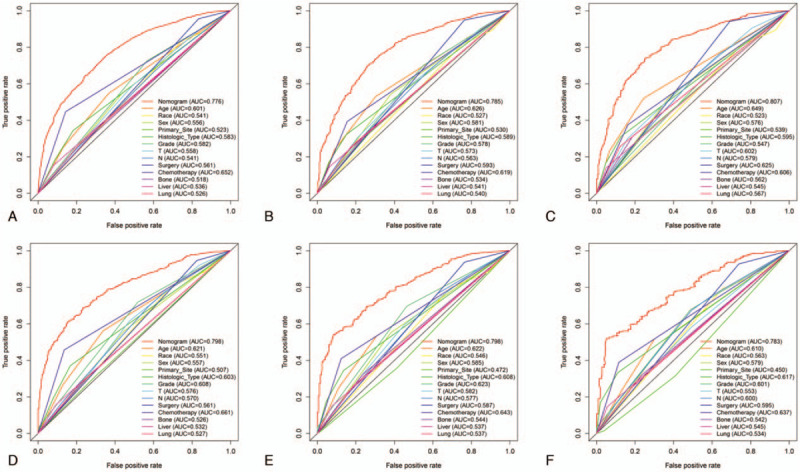
Comparison of prediction accuracy between nomogram model and independent prognostic factor. ROC curves of the nomogram for predicting the 1- (A), 2- (B) and 3-year (C) OS in the training cohort, and the 1- (D), 2- (E) and 3-year (F) OS in the validation cohort. OS = overall survival, ROC = receiver operating characteristic.

The accuracy of using the nomogram to predict the OS compared to predictions using a single independent prognostic factor has significant advantages, both in the training and validation cohorts (Fig. 3).

### 3.6 Evaluation of nomogram

**Figure 4:**
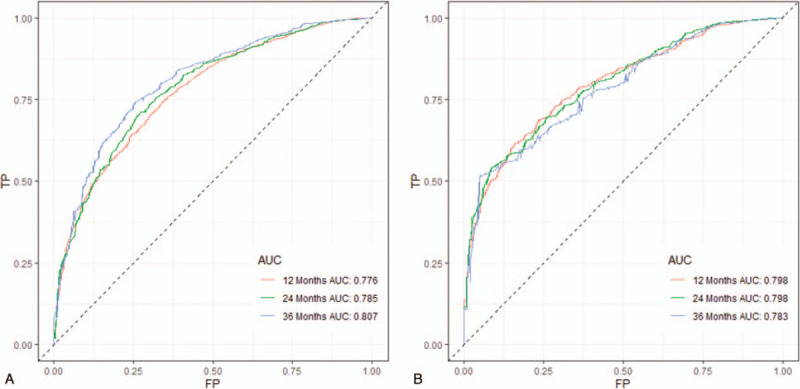
ROC curves. ROC curves for predicting 1-year, 2-year, and 3-year OS in the training cohort (A); ROC curves for predicting 1-year, 2-year, and 3-year OS in the validation cohort (B).

**Figure 5:**
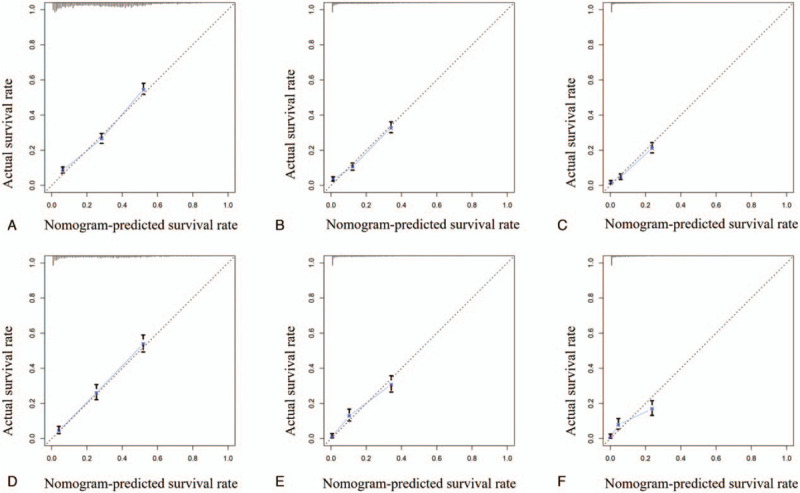
Calibration curves. The calibration curves of the nomogram for the 1-, 2-, and 3-year OS prediction of the training cohort (A–C), validation cohort (D–F).

**Figure 6:**
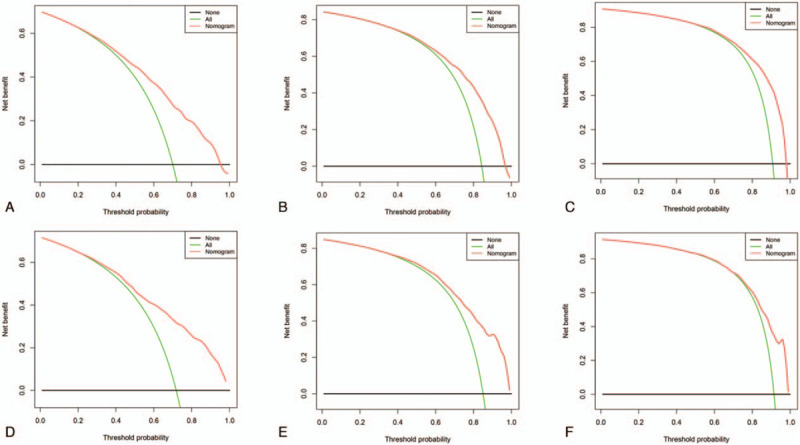
Decision curve analysis (DCA). DCA of the nomogram for predicting the 1- (A), 2- (B), and 3-year (C) OS in the training cohort, and the 1- (D), 2- (E), and 3-year (F) OS in the validation cohort.

**Figure 7:**
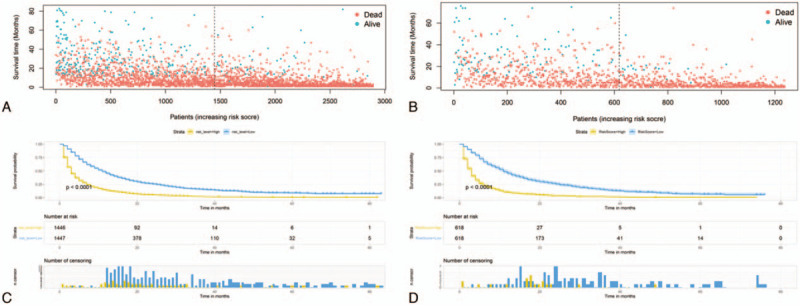
Kaplan–Meier survival analysis for both the training cohort and the validation cohort. Patients with a higher risk score demonstrated a worse prognosis than those with a low risk score in the training cohort (A, C) and validation cohort (B, D).

ROC curve showed that the area under curves of the clinical predictive model for the 1-, 2-, and 3-year OS reached 0.776, 0.785, and 0.807 in the training cohort; and 0.798, 0.798, and 0.783 in the validation cohort, respectively, which demonstrated a better discriminative ability (Fig. 4). The calibration curves for 1-, 2-, and 3-year OS demonstrated a strong agreement between actually observed probabilities and predicted probabilities (Fig. 5). The clinical application value of the nomogram was evaluated by DCA. AS shown in Fig. 6, DCA also showed that the nomogram had a good clinical utility in predicting OS in NSCLC patients with BM. Kaplan–Meier survival analysis of the signature for both the 2 cohorts. In the training and validation cohort, patients with higher risk scores demonstrated a worse prognosis than those with lower risk scores, suggesting the strong predictive ability for BM patient prognosis (Fig. 7).

## 4 Discussion

In this research, we construct a clinical predictive model to predict the OS of NSCLC with BM. A total of 4129 patients were enrolled, and 13 independent prognosis factors were identified by Cox regression analysis and incorporated to establish a clinical prediction model. The ROC curve indicated the clinical prediction model had a strong distinguishing ability. As is shown in Fig. 3, the model can predict 1-, 2-, and 3-year OS accurately. For all we know, this is the first study to construct a prognostic nomogram for NSCLC with BM based on a large and diverse case data. This predictive model can conveniently and directly predict the OS of patients and inform individuals about the benefits of some therapies, which is of great significance for clinical decision-making. We should note that not all surgeries can benefit patients.

This study found that age, race, sex, primary site, histologic type, grade, T stage, bone metastasis, surgery, chemotherapy, N stage, liver metastasis, and lung metastasis were the prognostic factors, which was consistent with the previous results.^[14]^ David et al^[15]^ found that the median OS of patients with surgically treated NSCLC with metastasis was significantly longer than those who received nonsurgical treatment (9.4–28 months vs. 2–10 months). However, it ought to be noticed that not all surgeries can benefit NSCLC patients with BM. We should decide whether to perform surgery on NSCLC patients with BM by considering therapy-related factors, disease-related factors, and careful multidisciplinary discussions.^[16]^ And the research found that the vast majority of those who underwent surgery experienced radiation therapy, most of whom experienced chemotherapy. Nevertheless, surprisingly, radiotherapy is not an independent prognostic factor (*P* = .075), which indicates radiotherapy has little effect on prognosis. Radiotherapy is currently considered an effective therapy for NSCLC. In a retrospective analysis study based on the SEER database, radiotherapy had been shown to significantly increase the OS of patients with IIIA/N2 NSCLC.^[17]^ It has also been reported that radiotherapy alone can prolong the median survival time of BM by 3 to 6 months. As we all know, the prognosis for BM is poor, with a median survival of less than 1 year. Due to the lack of standard therapy for patients with BM, patients often receive different treatments, including radiotherapy, surgery, individual systemic chemotherapy, and targeted therapy. Of course, the prognostic value of different treatments is still controversial.^[l7,18]^ Chemotherapy may still be an option for NSCLC patients with BM who are not suitable for targeted therapy or immunotherapy. Also, the traditional view is generally believed that due to the existence of the blood–brain barrier, the low pass rate of chemotherapeutic drugs, resulting in a limited therapeutic effect on intracranial metastases. Interestingly, our study shows that chemotherapy is a positive prognostic factor for patients. We believe that BM is mainly through the blood-derived pathway, so when patients undergo BM, the blood–brain barrier has been damaged to a certain extent, increasing the permeability to chemotherapy drugs, thereby improving the prognosis of patients.^[19,20]^ Therefore, radiotherapy combined with chemotherapy may be the most effective therapy for BM.

It has been reported that race is also closely related to the prognosis of LC, though this association is still controversial. A survey involving most Americans showed that blacks had lower survival rates than whites.^[21]^ Surprisingly, whites showed a more negative effect on prognosis than blacks in this study. Another 10-year study in the United States shows that differences in access to health care services cause racial differences in LC mortality.^[22]^ The International Staging Committee of the International Association for the Study of LC has published a paper about the impact of prognostic factors on NSCLC patients. Their findings indicate that histologic type is an important prognostic factor for NSCLC, and the prognosis of adenocarcinoma is better than other histologic types.^[23]^ In our results, adenocarcinoma is a positive prognostic factor for NSCLC with BM, which undoubtedly confirms the above view. Sperduto et al retrospectively analyzed the prognosis of 5067 patients who received BM treatment and found that the extracranial metastases were related to the prognosis of LC with BM.^[24]^ Our results suggest that liver metastasis in other distant metastatic sites is significantly associated with worse prognosis, followed by bone metastasis, which is highly consistent with the results of a previous study analyzing 17,431 LC patients.^[10]^

In previous studies, researchers based on clinical data analyzed factors related to the prognosis of NSCLC with BM. However, compared with the study of independent risk factors, the development of clinical prediction models is more meaningful for improving the prognosis of patients. More importantly, the indicators included in this study are all clinically easily obtained and determined indicators. Therefore, the model has better prediction ability and higher reliability, which can provide a reference for patient consultation, risk assessment, and clinical decision-making. It must be noted that this study also has certain limitations. First, this study was a retrospective study that included only patients with complete data, with inevitable deviations. Second, the SEER database provided limited information. This study did not take into account important prognostic factors that have been determined in previous research. Third, we do not have specific information about systemic treatment, especially the specific type of surgery, radiation dose, and choice of chemotherapy drugs. Fourth, the SEER database has only records of bone, brain, lung, and liver metastasis at the time of diagnosis, and metastases are not considered at follow-up. However, in this study, through a large sample data combined with rigorous statistical analysis, a series of factors related to the prognosis of NSCLC with BM were found and a prediction model was established, which is of great significance to both clinicians and patients.

## 5 Conclusion

We developed and validated a clinical prediction model to assess the individualized prognosis of NSCLC patients with BM. With this model, clinicians can estimate individual patient survival rates more accurately. We hope to promote the progress of personalized treatment through quantitative analysis of prognostic-related factors.

## Acknowledgments

The authors thank all the staff in Department of Minimally Invasive Spine Surgery, Affiliated Hospital of Chengde Medical University for their contribution on their research.

## Author contributions

Conceptualization: Zhangheng Huang, Chuan Hu, Chengliang Zhao.

Data curation: Zhangheng Huang, Zhiyi Fan.

Formal analysis: Zhangheng Huang, Yuexin Tong.

Methodology: Chuan Hu.

Supervision: Zhangheng Huang, Chengliang Zhao.

Writing – original draft: Zhangheng Huang.

Writing – review & editing: Zhangheng Huang, Chengliang Zhao.
